# Mapping the Evolution of Diagnostic Research on *Mycoplasma pneumoniae*: A Bibliometric Analysis (1980–2025)

**DOI:** 10.3390/pathogens15090912

**Published:** 2026-08-29

**Authors:** Mo Wu, Jun Wang, Zhen Xie, Yun Xiang, Cong Yao, Mei Liu, Yu Shang, Chengyu Li, Xiang Ma, Wenbin Tuo, Hui Du, Lanxiang Huang, Qinzhen Cai

**Affiliations:** 1 Department of Laboratory Medicine, Wuhan Children’s Hospital (Wuhan Maternal and Child Healthcare Hospital), Tongji Medical College, Huazhong University of Science & Technology, Wuhan 430016, China; wumo@zgwhfe.com (M.W.); sywj0928@126.com (J.W.); xiangyun5272008@163.com (Y.X.); 13477000850@163.com (Y.S.); maxiang@zgwhfe.com (X.M.); tuowenbin@zgwhfe.com (W.T.); 2 School of Public Health, Wuhan University of Science and Technology, Wuhan 430065, China; li-chengyu@wust.edu.cn; 3 Department of Laboratory Medicine, The Fifth Hospital of Wuhan, Wuhan 430050, China; xiezhen9526@163.com; 4 Health Care Department, Wuhan Children’s Hospital (Wuhan Maternal and Child Healthcare Hospital), Tongji Medical College, Huazhong University of Science & Technology, Wuhan 430016, China; yaocsmart@163.com; 5 Department of Laboratory Medicine, Wuhan Hankou Hospital, Wuhan 430012, China; sywj0928@163.com; 6 Department of Respiratory Medicine, Wuhan Children’s Hospital (Wuhan Maternal and Child Healthcare Hospital), Tongji Medical College, Huazhong University of Science & Technology, Wuhan 430016, China; 7 Department of Clinical Laboratory, The First Affiliated Hospital of Guangxi Medical University, Key Laboratory of Clinical Laboratory Medicine of Guangxi Medical University, Nanning 530021, China

**Keywords:** *Mycoplasma pneumoniae*, diagnosis, bibliometric analysis, polymerase chain reaction, metagenomic next-generation sequencing, predictive model

## Abstract

*Mycoplasma pneumoniae* (MP) is a major etiological agent of respiratory tract infections. This study sought to systematically map the current landscape, thematic progression, collaborative networks, and emerging priorities in diagnostic research on MP infections. Data were extracted from the Web of Science Core Collection from 1 January 1980, to 12 May 2025. Bibliometric and visualization analyses across countries/regions, institutions, authors, co-cited references, keywords, and disease-related terms were conducted using CiteSpace, VOSviewer, Pajek, and SCImago Graphica. Analysis of 2093 articles revealed consistent growth in research output related to MP diagnosis. Most publications originated in China (*n* = 623). The United States Centers for Disease Control and Prevention demonstrated the greatest total link strength in institutional collaboration networks. Key high-frequency keywords reflect the ongoing transition from traditional pathogen confirmation to integrated diagnostic approaches incorporating molecular testing, macrolide-resistance detection, co-infection evaluation, and risk stratification. Current research has increasingly targeted diagnostic optimization and the early identification of refractory or severe *Mycoplasma pneumoniae* pneumonia. This study provides a structured overview of the knowledge structure, thematic evolution, and technological frontiers in MP diagnostic research. The findings highlight key challenges, thereby offering guidance for interdisciplinary innovation and informing future diagnostic research directions.

## 1. Introduction

*Mycoplasma pneumoniae* (MP) is a major causative pathogen of respiratory infections, accounting for 10–40% of community-acquired pneumonia (CAP) cases in children, making it a substantial global health concern [1,2]. Although most MP infections are mild, the incidence of severe cases has risen, resulting in pulmonary and extrapulmonary complications, long-term sequelae, and even death [3,4,5,6]. Treatment is complicated by increasing rates of macrolide-resistant MP—averaging 81% (95% confidence interval [CI] 73–87%) in China over the past 20 years [7] and reaching 93.02% in 2024 [1]. This highlights the requirement for timely and accurate diagnosis.

Currently, several methods are used for the primary diagnosis of MP infections, including direct culture, serological testing, and nucleic acid detection. Each approach has certain limitations: culture, although highly specific, is time-consuming [8,9]; serology may yield false-negative results or fail to distinguish between past and current infections [10,11]; and nucleic acid-based methods, such as real-time polymerase chain reaction (RT-PCR), are sensitive but cannot confirm bacterial viability, raising questions regarding the clinical relevance of positive results [12,13]. Recent advancements in molecular biology have enabled the clinical application of innovative detection methods, including multiplex PCR, loop-mediated isothermal amplification (LAMP), droplet digital PCR, and metagenomic next-generation sequencing (mNGS). Although these techniques markedly improve detection sensitivity and reduce the turnaround time [14,15,16,17], their application is limited by the cost of equipment and operational complexity [16]. Beyond direct pathogen detection, predictive models based on blood biomarkers [18] and multimodal strategies integrating clinical indicators with imaging modalities, such as lung ultrasound [19] and chest computed tomography (CT) [20], have also been explored to support the diagnosis and clinical assessment of *Mycoplasma pneumoniae* pneumonia (MPP). Taken together, these advances underscore the value of using bibliometric analysis to examine nearly five decades of MP laboratory diagnostic research. This approach provides insight into the evolution and clinical application of diagnostic technologies, as well as the historical development and future trends of MP diagnosis and management.

The current study applied bibliometric methods to map the evolution of MP diagnostic research from 1980 to 2025. Using data derived from the Web of Science Core Collection (WoSCC), as well as analytical tools such as VOSviewer and CiteSpace, it examined the following: (1) technological transitions from culture and serology to multi-omics and multimodal diagnostics; (2) key literature and knowledge clusters influencing the field; and (3) emerging research directions and persistent clinical challenges, including macrolide resistance and early identification of severe cases. By integrating publication trends, co-authorship networks, co-citation patterns, and keyword usage over time, this study transcends descriptive statistics to explore the development of MP diagnostics. It identified current topics, influential contributors, collaboration pattern, and underexplored areas that could guide future innovation. Additionally, a knowledge map was developed to guide research priorities, foster interdisciplinary collaboration, and support evidence-based decision-making for precision diagnosis and treatment. By organizing diverse studies into a cohesive framework, this method could help researchers, clinicians, and policymakers navigate MP diagnostics and anticipate emerging challenges and opportunities.

## 2. Materials and Methods

### 2.1. Data Source and Search Strategy

The WoSCC was used as the data source in this study because its rigorous journal selection and data curation processes provide standardized and carefully curated bibliographic metadata and cited-reference data suitable for citation-based bibliometric analyses. The WoSCC was selected as the sole data source to maintain consistency in document indexing, citation links, and metadata, and avoid duplication and heterogeneity arising from cross-database integration. The following topic search strategy was applied: TS = (“Mycoplasma pneumoniae”) AND TS = (detection OR testing OR diagnosis). The literature search covered all publications in the database from its inception through 12 May 2025, with systematic retrieval completed on 12 May 2025. The initial search yielded 2648 documents.

### 2.2. Inclusion and Exclusion Criteria

The inclusion criteria were as follows: (1) publication date from 1 January 1980 to 12 May 2025; (2) peer-reviewed original journal articles; (3) English-language publications; and (4) relevance to diagnosis for MP. Excluded publications comprised reviews, letters, editorials, meeting abstracts, and book reviews. Gray literature, including dissertations and reports, was also excluded, as were preprints. Two independent reviewers screened the titles and abstracts for eligibility, and any disagreements were resolved through discussion with a third researcher until consensus was reached.

**Figure 1:**
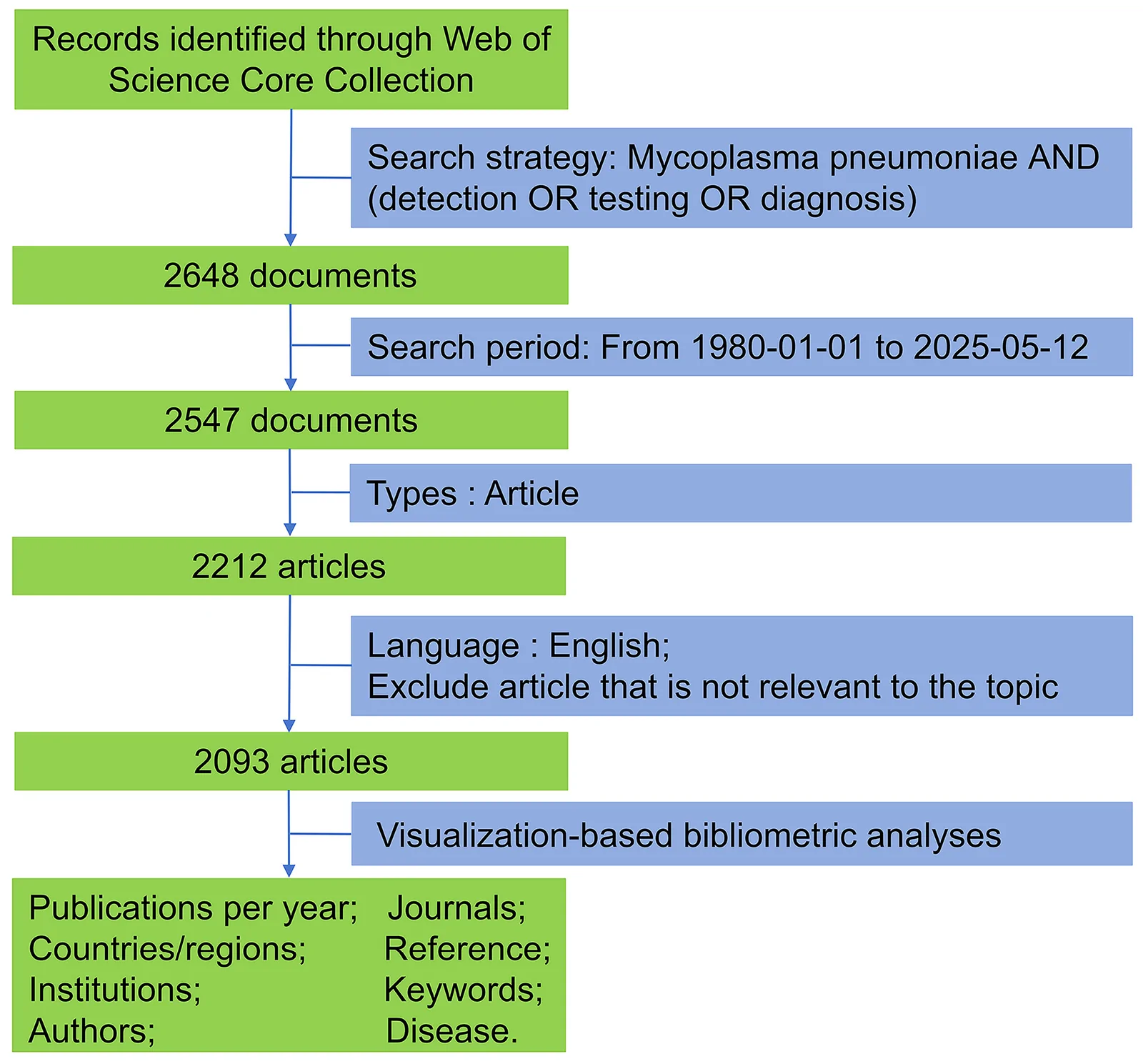
Literature search strategy and screening process flowchart.

Ultimately, 2093 records constituted the final dataset (Figure 1). Metadata, including countries/regions, institutions, authors, journals, cited references, and keywords, were extracted for bibliometric analyses. Disease associations were visualized using the Citexs platform (https://www.citexs.com, accessed on 23 May 2026).

### 2.3. Bibliometric Analysis

Before analysis, synonyms and spelling variants were standardized across author names, institution names, country/region names, and keyword variants. A multilayered analytical framework characterized the intellectual landscape of MP diagnostic research. Co-authorship networks and keyword co-occurrence networks were constructed using VOSviewer (version 1.6.18) [21]. Pajek (version 5.16) was used to refined network layouts, while SCImago Graphica (version 1.0.35) facilitated visualization of country/region collaboration patterns on geographical maps. CiteSpace (version 6.3.R1) [22] was employed to identify co-citation clusters and citation bursts. Betweenness centrality was also calculated for network nodes, with values > 0.10 used to identify nodes with high betweenness centrality [23]. The Pathfinder algorithm and timeline views provided insights into the temporal evolution of major research themes.

In the network maps, node size represented citation or occurrence frequency, whereas link thickness denoted co-citation or collaboration strength. CiteSpace visualizations used color coding to distinguish temporal evolution and thematic differentiation, highlighting structural hubs with rose-pink rings.

### 2.4. Statistical Analysis

As the literature search concluded on 12 May 2025, records from 2025 were excluded from Compound Annual Growth Rate (CAGR) calculations and polynomial regression modeling, which were performed using complete-year data spanning 1980 to 2024. Quadratic polynomial regression was used to model cumulative publication trends in Microsoft Excel 2021. The CAGR of the annual publication output was calculated using Equation (1) [24]:

(1) CAGR=Number of publications in 2024Number of publications in 19801n−1×100

where *n* denotes the number of growth intervals between the initial (starting) and final years (44 in this analysis).

Heat maps and circular plots were generated using R package ComplexHeatmap (version 2.16.0; https://github.com/jokergoo/ComplexHeatmap (accessed on 23 May 2026) and R package circlize (version 0.4.15) [25]. The quality of the clustering structure was evaluated using modularity Q and mean silhouette S.

## 3. Results

### 3.1. Analysis of Annual Publication Trends and Geographic Contributions

Publications on MP diagnostics demonstrated a consistent upward trend from 1 January 1980 to 12 May 2025. Based on complete calendar-year data from 1980 to 2024, the annual publication output increased from 12 articles in 1980 to 141 in 2024, corresponding to a CAGR of 5.76%.

**Figure 2:**
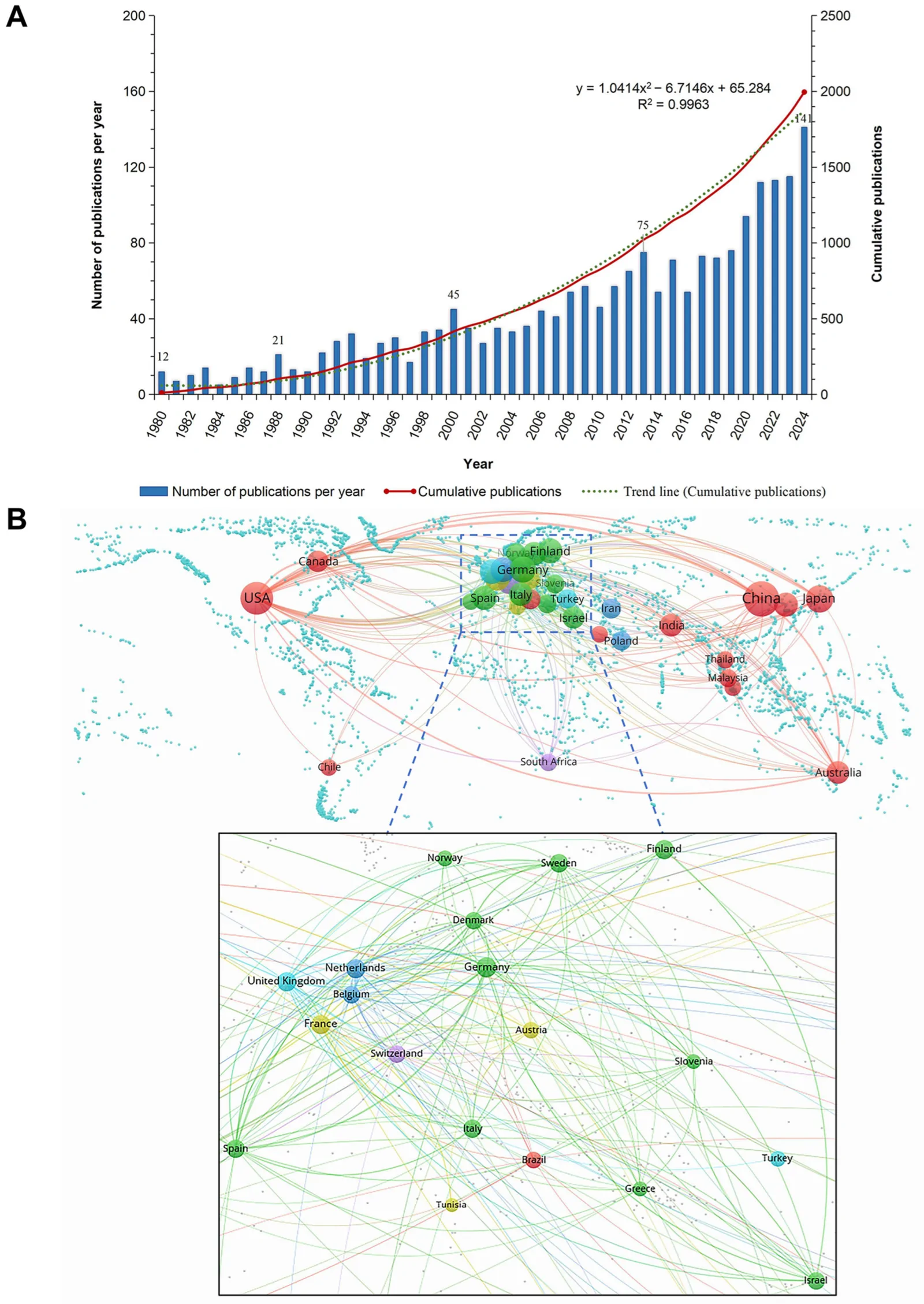
Annual publication trends and country/region collaboration network. (**A**) Annual and cumulative publication trends in the field. Blue bars indicate annual publication counts on the left y-axis, whereas the red curve indicates cumulative publication counts on the right y-axis. Polynomial regression was fitted exclusively to cumulative publication data from complete calendar years between 1980 and 2024. In the model, *x* represents the coded year (*x* = year − 1979), and y represents the cumulative number of publications. Records from 2025 were excluded from model fitting as the literature search was completed on 12 May 2025, and did not cover the full calendar year. (**B**) Country/region collaboration network. Countries/regions with at least eight publications were included. Each node represents a country or region; node size is proportional to publication output, node colors indicate clusters within the collaboration network, and edges represent collaboration between countries/regions, with edge thickness indicating the strength of collaboration.

To further quantify the temporal evolution of the literature, quadratic polynomial regression was performed between the cumulative publication count and year using data from 1980 to 2024, yielding the following model: *y* = 1.0414*x*^2^ − 6.7146*x* + 65.284, where *x* represents the coded year (*x* = Year − 1979) and *y* denotes cumulative publications (*R*^2^ = 0.9963) (Figure 2A). From 2021 to 12 May 2025, China, Bangladesh, Malaysia, Nepal, Pakistan, Romania, and Singapore emerged as emerging countries of origin (Figure S1A). Among these, China exhibited the most rapid growth in publication output, ranking first globally with 623 publications (Table 1 and Figure 2B).

The country/region burst analysis revealed sustained early citation bursts in Finland and England, while China exhibited the strongest recent burst (strength: 132.04, 2021–2025), indicating a marked increase in recent publication activity (Figure S1B). Within the country/region co-authorship network, the United States achieved the highest total link strength (TLS = 138), indicating extensive international collaboration (Table 1). The Netherlands and Belgium displayed the strongest bilateral collaboration, with a normalized link strength of 18 (Table S1). Overall, these findings suggest a transition from early research activity mainly observed in Europe toward a more global collaboration framework encompassing Asia, North America, and Europe.

### 3.2. Analysis of Research Institutions

**Figure 3:**
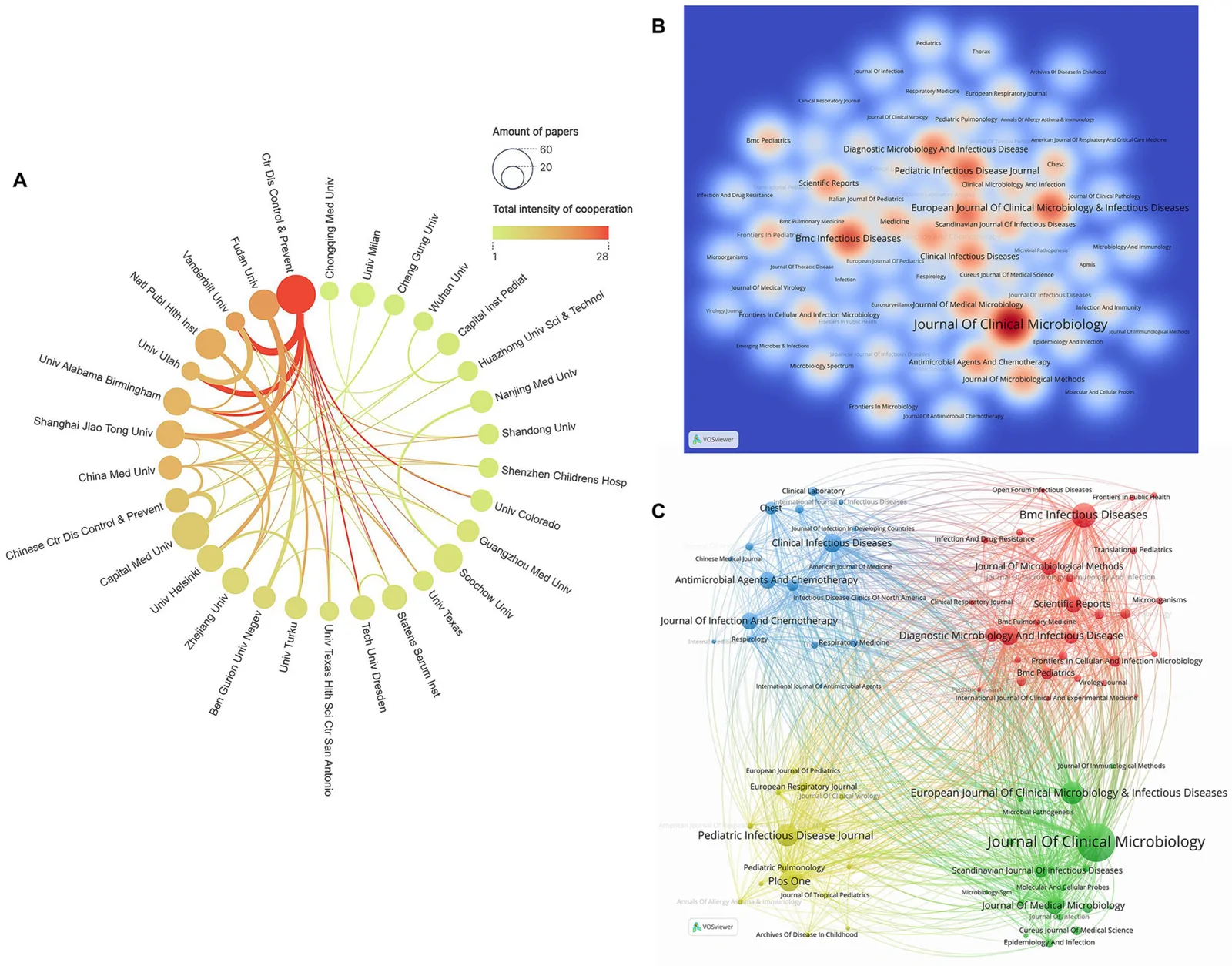
Institutional collaboration, journal activity, and journal publication clusters. (**A**) Institutional collaboration network. Institutions with at least 12 publications were included. Each node, consisting of a circle and its label, represents an institution. Node size is proportional to the number of publications, and edge thickness indicates the strength of collaboration between institutions. The color gradient represents the total collaboration strength of each institution with other institutions. (**B**) Journals with at least seven publications were included. Each circle–label pair represents a journal. Circle size represents the number of publications, and color intensity reflects the relative concentration of journal publication activity. (**C**) Journal publication clustering network. Journals with at least five publications were included. Each circle–label pair represents a journal. Circle size is positively associated with journal publication output, and edge thickness indicates link strength between journals. Colors indicate clusters identified based on the journal network. Four journal clusters are shown in red, green, blue, and yellow.

Analysis of 2686 research institutions identified the United States Centers for Disease Control and Prevention (U.S. CDC) as the leading entity in research output (*n* = 54, Figure 3A and Table S2) as well as the institution with the most extensive collaborative network (total intensity of cooperation = 28; Table S2). The most significant partnership was observed between the U.S. CDC and Vanderbilt University, exhibiting a normalized link strength of 9 (Table S2). Citation burst analysis delineated institutional influence into two distinct phases. The National Institutes of Health (NIH) demonstrated the highest burst strength (12.27) over the longest duration (1982–2005), whereas the U.S. CDC showed a later but substantial burst (11.48) from 2012 to 2019 (Figure S2A). Notably, seven Chinese universities experienced concentrated citation bursts between 2020 and 2025, indicating a recent increase in visibility and research activity among East Asian institutions. These temporal variations highlight the ongoing geographical redistribution of research productivity within the global institutional landscape.

### 3.3. Journal Analysis

The analysis of journal publications revealed notable temporal changes in the research landscape. Initial research outputs were predominantly published in the *Journal of Infectious Diseases* and the *Journal of Clinical Pathology*, with the former demonstrating the highest citation-burst strength (63.04; burst duration: 1983–2008) and the latter exhibiting the longest sustained burst (1981–2010). Thereafter, citation bursts became more prominent in open-access journals, including *Frontiers in Microbiology*, *Frontiers in Cellular and Infection Microbiology*, and *Scientific Reports* (Figure S2B). Of the 626 journals surveyed, the *Journal of Clinical Microbiology* published the largest number of relevant articles (*n* = 100), reaching peak publication activity in 2004–2005. This was followed by *BMC Infectious Diseases* (*n* = 54), which demonstrated heightened activity during 2019–2020 (Figure 3B and Table S3). Cluster analysis categorized these journals into four main clusters: laboratory diagnostic techniques and microbial methodology; anti-infective pharmacotherapy and pulmonology; clinical microbiology and infectious disease surveillance; and pediatric infectious diseases and general clinical research (Figure 3C).

### 3.4. Author Collaboration and Reference Co-Citation Analysis

**Figure 4:**
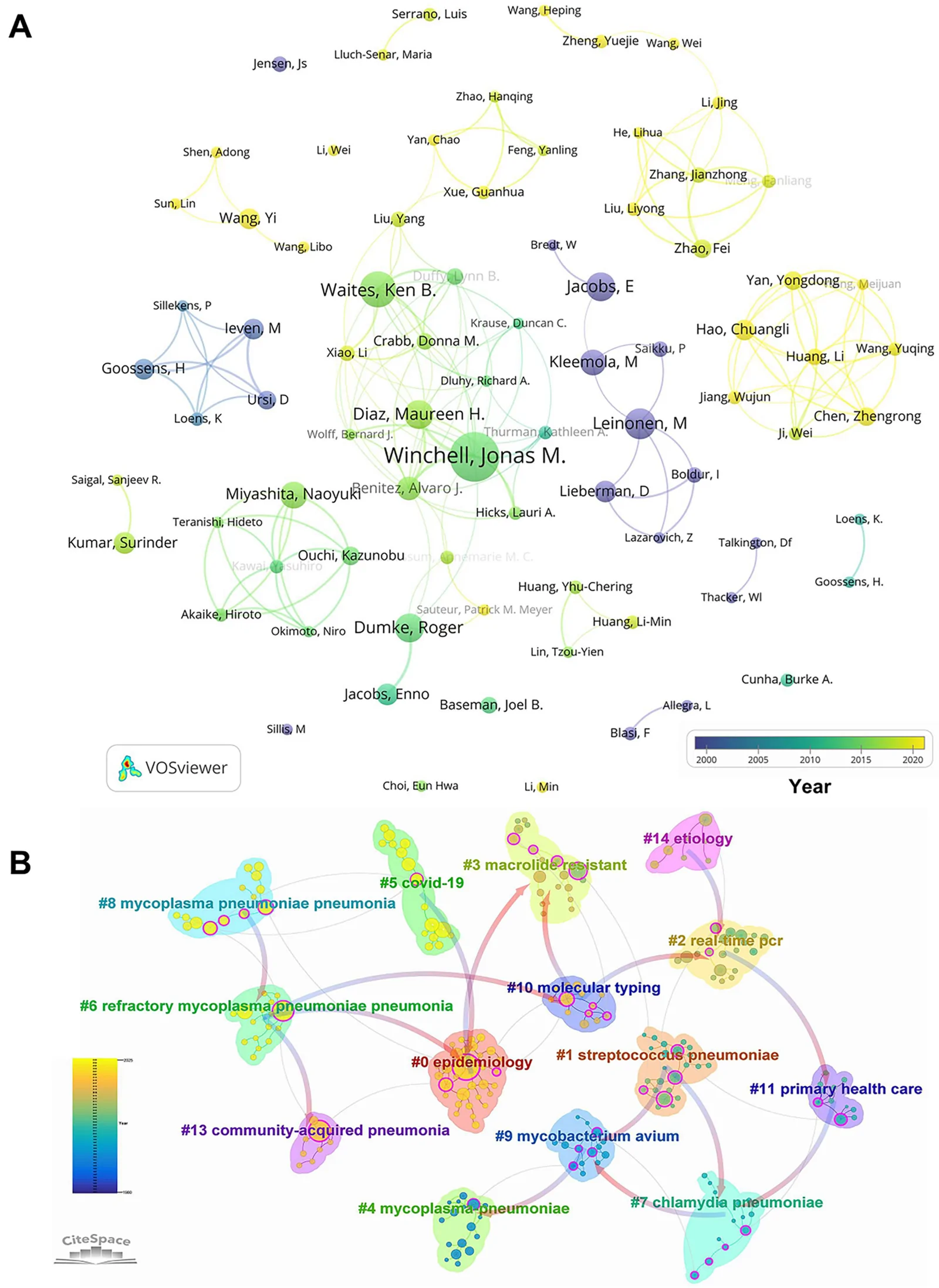
Author collaboration network and article co-citation clusters. (**A**) Author collaboration network with a temporal overlay. Authors with at least seven publications were included. Each node, consisting of a circle and its label, represents an author. Node size is proportional to the number of publications, and edge thickness indicates the strength of collaboration between authors. Node color indicates the average publication year according to the color scale in the lower right, with blue representing earlier average publication years and yellow representing more recent years. (**B**) Article co-citation clustering network. Time slicing was set from 1980 to 2025, with one year per slice and a selection criterion of *k* = 2. Colored cluster regions and their labels indicate thematic clusters generated based on article co-citation patterns. Edges represent co-citation relationships among the included references. Nodes with rose-pink outer rings were identified as pivotal references with centrality greater than 0.1. Network clustering yielded a Modularity Q of 0.8828 and a Mean Silhouette S of 0.9709.

Analysis of 10,890 authors highlighted Winchell JM as the most prolific contributor (29 articles), with a particularly strong collaborative relationship with Diaz MH (normalized link strength = 16; Table S4). Notable contributions included Waites KB (21 articles) and Leinonen M (18 articles; Table S4). Temporal mapping indicated that Jacobs E, Kleemola M, and Leinonen M were active before 2000, succeeded by Dumke R and Winchell JM between 2011 and 2015, whereas Diaz MH and Kumar S reached their publication peaks around 2016 (Figure 4A). The top 10 cited authors exhibited citation bursts primarily from 2003 to 2018 (Figure S3A). Winchell JM recorded a maximum burst strength (11.11) over 11 years (2008–2018), while Waites KB and Benitez AJ experienced the most recent burst onset in 2014 (Figure S3A).

Co-citation mapping identified several high-impact nodes, such as Waites KB (2017) [26], Jain S (2015) [27], and Kutty PK (2019) [28] (Figures S2C and S3B). Citation bursts associated with Kutty PK (2019) [28] and Li ZJ (2021) [29] persisted through 2025, indicating continued citation momentum (Figure S2C). Clustering analysis revealed 15 thematic groups (Figure 4B); notably, Clusters #2 (RT-PCR) and #10 (molecular typing) functioned as key methodological bridges connecting distinct research domains. These clusters exhibited dense co-citation links connecting the domains of foundational pathogen identification (#14 etiology) and antimicrobial-resistance profiling (#3 macrolide resistance). The network topology suggests a progressive shift in research focus—from general etiological detection toward more refined characterization of resistance mechanisms and severe clinical phenotypes—as evidenced by close interconnections among clusters related to macrolide resistance (#3), refractory mycoplasma pneumoniae pneumonia (#6), real-time pcr (#2), and molecular typing (#10).

### 3.5. Keyword Co-Occurrence and Clustering

**Figure 5:**
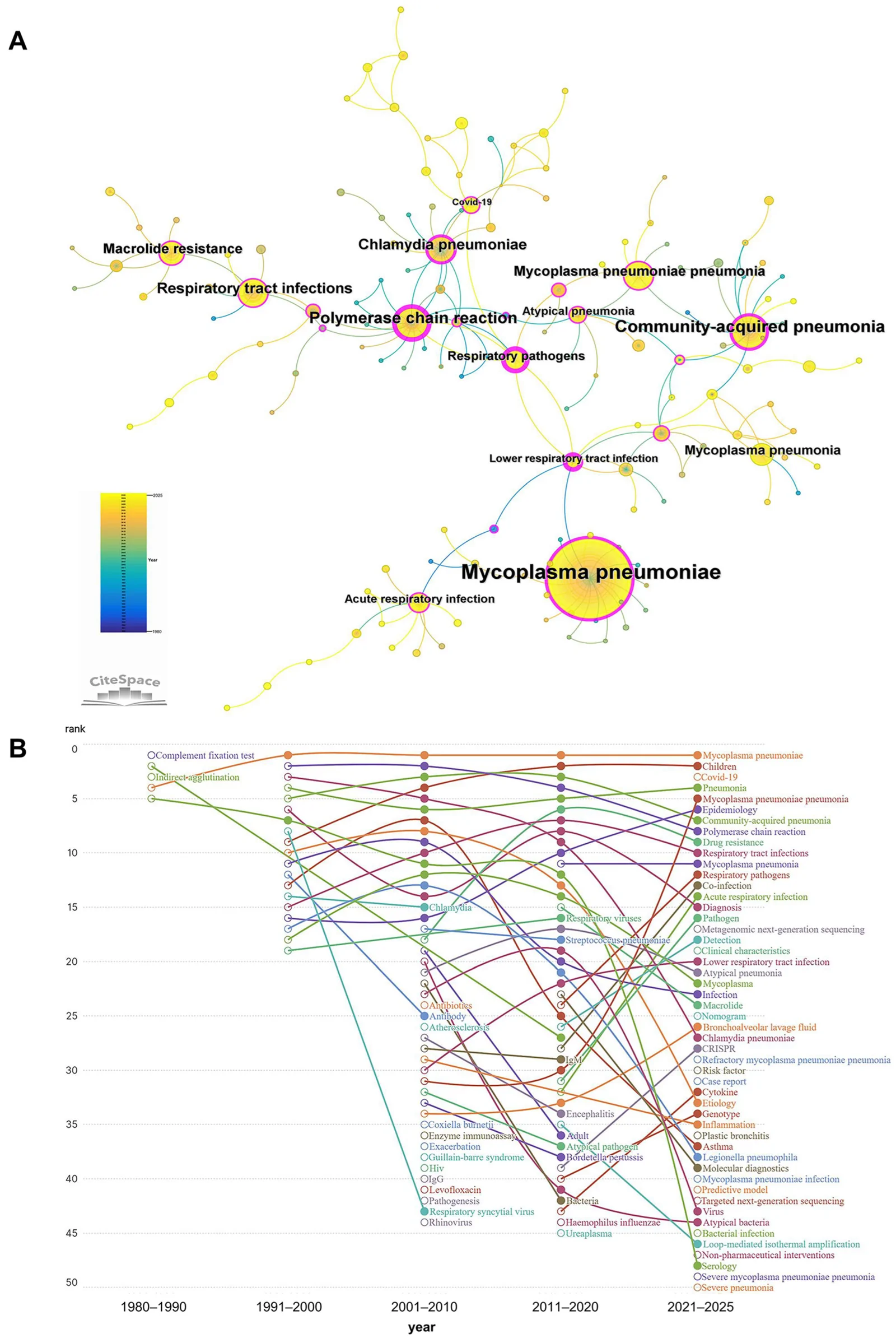
Keyword co-occurrence network and temporal rank evolution. (**A**) Keyword co-occurrence network. CiteSpace was run with a selection criterion of *k* = 6. Each node represents a keyword. The overall node size, formed by superimposed annual rings, is proportional to keyword frequency. Node colors indicate the years in which the keywords appeared: purple (earlier occurrences), yellow (more recent occurrences); multicolored annual rings indicate that a keyword appeared in multiple corresponding years. Edges represent co-occurrence relationships between keywords. Nodes with rose-pink outer rings indicate pivotal keywords in the co-occurrence network. (**B**) Temporal rank evolution of the top 50 keywords. Keywords were grouped into five periods: 1980–1990, 1991–2000, 2001–2010, 2011–2020, and 2021–2025, and ranked by frequency within each period. Lower rank values indicate higher keyword prominence. Curve fluctuations show the changes in keyword popularity rankings over time. Open and solid circles indicate the first and last observed periods of each keyword, respectively.

Keyword co-occurrence analysis revealed that “MP” (534 occurrences), “CAP” (160), and “polymerase chain reaction (PCR)” (127) were the most frequently appearing nodes (Figure 5A and Table S5). These keywords occupied central positions within the network, with “PCR” demonstrating the highest betweenness centrality among the three keywords (0.48), underscoring its role as a major methodological bridge (Table S5). Burst analysis indicated that “MPP” attained a burst strength of 14.77 from 2022 to 2025. Concurrently, “acute respiratory infection” exhibited a sustained burst between 2020 and 2025, aligning with the post-COVID-19 surveillance period (Figure S2D).

Trend analysis of the top 50 keywords highlighted the evolution in technological emphasis. Research conducted from 1980 to 2000 primarily focused on serological testing, including the “complement fixation test” and “serology.” The subsequent decade (2001–2010) witnessed an increase in nucleic acid detection options, establishing “PCR” as the predominant methodological node. Between 2011 and 2020, research focus broadened to encompass molecular epidemiology, as evidenced by the emergence of terms such as “drug resistance” and “genotype.” More recently, from 2021 to 2025, terms such as “mNGS,” “tNGS,” “nomogram,” “predictive model,” and “clustered regularly interspaced short palindromic repeats (CRISPR)” emerged (Figure 5B), reflecting diversification in detection strategies.

**Figure 6:**
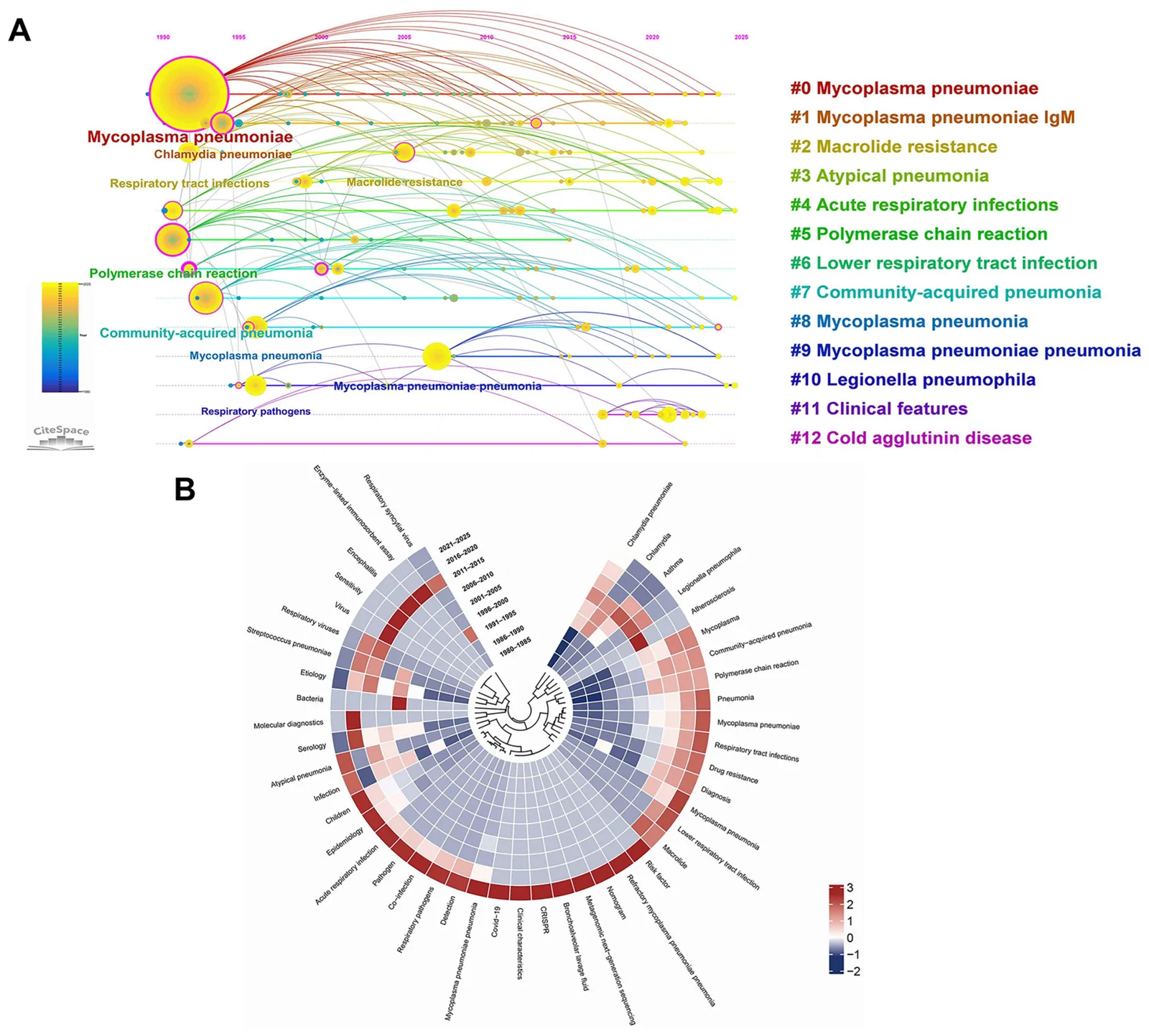
Keyword cluster timelines and temporal frequency heatmap. (**A**) Timeline visualization of keyword clusters. Keywords were clustered according to their co-occurrence relationships and arranged along horizontal timelines, with keywords from the same cluster placed on the same line. The time point of first keyword occurrence is shown at the top of the view, with more recent years positioned further to the right. The overall node size, formed by superimposed annual rings, is proportional to keyword frequency. Edges represent co-occurrence relationships between keywords. Node colors indicate the years in which the keywords appeared: purple (earlier occurrences), yellow (later occurrences); multicolored rings indicate that a keyword appeared in multiple corresponding years. Nodes with rose-pink outer rings indicate high-centrality keywords that served as pivotal points in the network. The number of keywords and the temporal span within each cluster reflect the relative prominence and duration of the corresponding research theme. (**B**) Temporal heatmap of keyword frequency. Keywords are arranged around the outer ring, and the concentric segments represent different publication periods. Color intensity represents the relative keyword frequency on the scale, with warmer colors indicating higher frequency and cooler colors indicating lower frequency.

Visualization of keyword clusters over time identified 13 major thematic clusters (Figure 6A). Earlier themes emphasized early screening (#1 MP IgM) and pathogen identification (#5 PCR). Subsequent clusters reflected a transition toward clinical management guidance, particularly #2 Macrolide resistance. Integrated syndromic frameworks, including #4 Acute respiratory infections and #3 Atypical pneumonia, were incorporated into broader differentiation networks involving #10 *Legionella pneumophila*.

The keyword frequency heatmap outlined the phasic evolution of the research foci over four decades (Figure 6B). From 1980 to 2000, the emphasis was on fundamental etiology and pathogen differentiation, with frequent references to “respiratory syncytial virus” and “chlamydia.” Between 2006 and 2015, attention shifted toward diagnostic challenges, including “drug resistance” and “CAP.” In the final interval (2021–2025), new high-density nodes appeared, including “risk factor,” “mNGS,” “nomogram,” “clinical characteristics,” “co-infection,” and “refractory *Mycoplasma pneumoniae* pneumonia (RMPP)” (Figure 6B). Collectively, these patterns signal a progressive shift in research priorities from basic etiological identification toward severity prediction, co-infection assessment, resistance management, and broad-spectrum molecular screening.

### 3.6. Disease-Related Terms in MP Diagnostic Research

**Figure 7:**
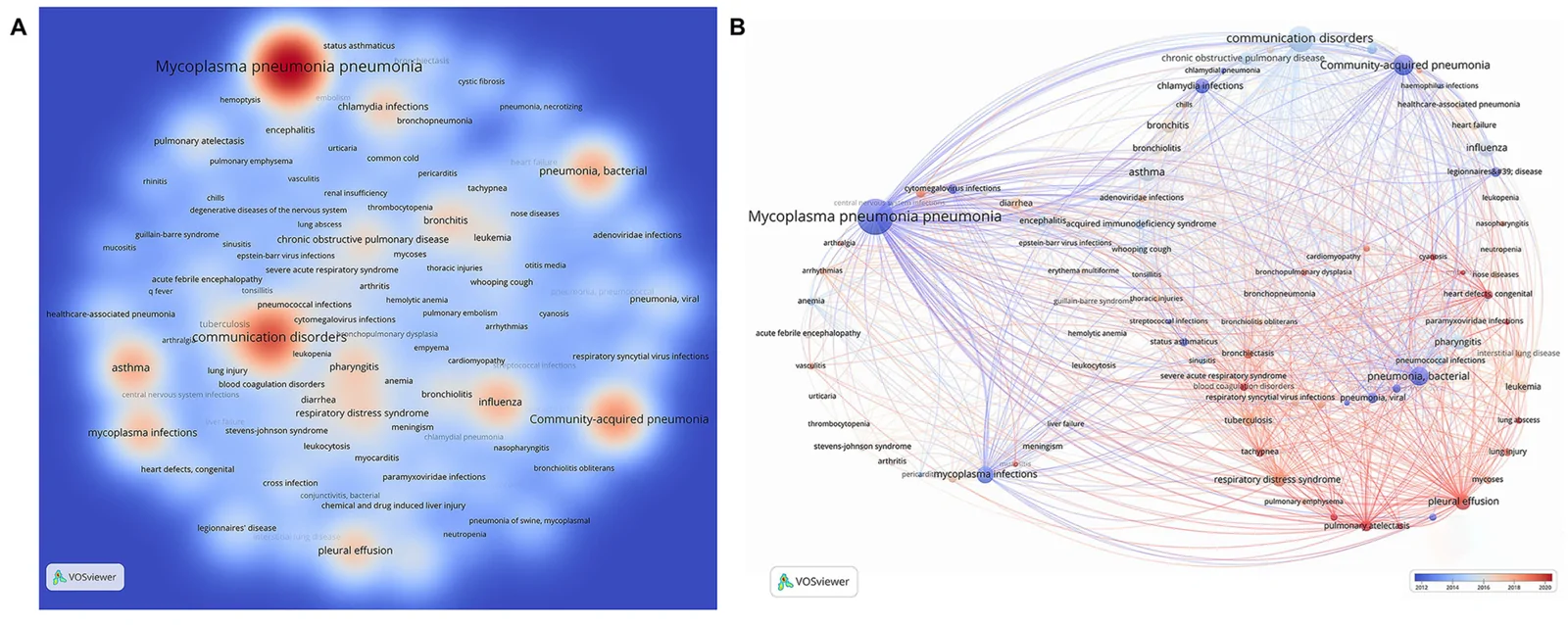
Associated disease-term frequency and temporal evolution. (**A**) Heatmap of associated disease-term frequency. A total of 1537 associated disease terms were included, with a minimum occurrence threshold of 20. Color intensity indicates the relative frequency or concentration of disease-related terms in the analyzed literature. (**B**) Temporal overlay visualization of associated disease terms. The inclusion criteria and occurrence threshold were the same as those in panel A. Each node, consisting of a circle and its corresponding label, represents an associated disease term. Node size is proportional to the frequency of the disease term. Node color indicates the average year of occurrence according to the color scale in the lower right, with blue representing earlier average occurrence years and red representing more recent years.

A heatmap analysis of disease-related terms revealed MPP, CAP, bacterial pneumonia, communication disorders, and asthma as the most frequently studied diseases (Figure 7A). Time-zone analysis demonstrated that MPP, CAP, and bacterial pneumonia were predominant research topics from 2010 to 2012. After 2018, the focus transitioned to severe complications, including pleural effusion, pulmonary atelectasis, and respiratory distress syndrome (Figure 7B). This progression reflects the evolution in research priorities from pathogen detection toward severe clinical phenotypes, complications, co-infections, and early stratification of disease progression. These findings highlight the increasing clinical demand for diagnostic systems capable of predicting disease severity and guiding individualized management strategies.

## 4. Discussion

This bibliometric study presents a systematic analysis of the progression of diagnostic research on MP from 1980 through 12 May 2025. By integrating publication trends, collaboration networks, co-citation clusters, keyword evolution, and disease-related terminology, this study offers a structured knowledge synthesis extending beyond basic descriptive visualization. The findings demonstrate the development of MP diagnostic research in response to evolving clinical demands and advances in diagnostic technology, while also identifying areas requiring additional investigation.

### 4.1. Publication Trends and Growth Characteristics

Over the past four decades, the publication trajectory for MP diagnostic research has exhibited sustained growth, with a CAGR of 5.76%. A quadratic polynomial model fitted to cumulative publication counts yielded a high coefficient of determination (R^2^ = 0.9963), indicating that the cumulative publication trajectory was well described by a nonlinear upward trend. This growth pattern reflects not only increasing publication output but also growing attention to MP diagnostics research. Notable surges in publication activity during the early and late 1990s may correspond with documented MP epidemic cycles [30,31] and the introduction of PCR-based diagnostic approaches [32,33]. Recently, the resurgence of MP following the COVID-19 pandemic [34] and the rapid rise of macrolide-resistant MP [1] have underscored the need for prompt, clinically actionable diagnostics that facilitate both pathogen identification and detection of resistance genes [1]. These observed publication trends are possibly the result of the dynamic interplay between public health needs and advances in diagnostic technologies.

### 4.2. Country/Region and Institutional Collaboration Patterns

Analysis of collaboration patterns revealed a clear mismatch between publication output and network centrality, emphasizing that publication volume does not necessarily reflect collaborative strength. Although most published articles originated in China, its average citation impact and collaboration link strength were lower than those of the United States, which continued to serve as a major intellectual hub of MP diagnostic research, with a relatively high citation influence [26,35]. The strong Netherlands–Belgium partnership further demonstrates the importance of regional collaboration within Europe. Overall, research in this area is now more geographically diverse, with rapidly increasing their contributions from Asian countries, while collaborative links predominantly remain focused around North American and European institutions. Future research should therefore prioritize coordinated international collaboration by adopting shared case definitions, standardized sampling procedures, comparable diagnostic platforms, and harmonized resistance-surveillance frameworks.

At the institutional level, the centrality of the CDC and NIH reflects their long-standing roles in surveillance, diagnostic standardization, and method development. Their contributions to molecular typing, macrolide-resistance monitoring, and multicenter diagnostic studies have made them the central hubs of global collaboration efforts [36,37]. Chinese universities, including Shanghai Jiao Tong University and Capital Medical University, have become increasingly active in developing diagnostic technologies and translating them into clinical practice [10,38], highlighting East Asia’s growing influence in this field. However, to ensure that new diagnostic tools are applicable beyond single centers or regions, increased research output must be accompanied by stronger cross-institutional collaboration, external validation of diagnostic models, and transparent data-sharing practices.

### 4.3. Evolution of Knowledge Carriers and Research Themes

The distribution of source journals reflects the evolving disciplinary landscape of MP diagnostic research. Earlier investigations were concentrated on clinical microbiology and infectious disease journals, which were major publication venues for culture-based, serological, and PCR-based detection techniques [39,40]. More recent publications have increasingly appeared in journals focused on molecular diagnostics, infectious disease surveillance, respiratory medicine, pediatrics, and open-access multidisciplinary platforms [20,41,42,43,44]. This progression reflects the gradual expansion of MP diagnostic research from laboratory-scale microbiology to broader clinical and multidisciplinary domains, providing a disciplinary context for the historical development and clinical application of diagnostic technologies. Analysis of author collaboration patterns further underscores this evolution: early influential works laid the foundations of serological testing and pathogen identification, whereas subsequent research groups have advanced real-time PCR, molecular typing, detection of macrolide resistance, and drug-susceptibility monitoring [45,46,47]. Together, these journal and author patterns provide historical and collaborative context for the evolution of MP diagnostic research and help indicate where research activity and expertise have become concentrated, thereby complementing the thematic analyses used to identify current research directions and providing context for the research capacity available to advance these priorities.

The reference co-citation network, characterized by a high modularity Q value and mean silhouette S value, revealed a well-structured clustering of intellectual communities rather than isolated research topics. Notably, Clusters #2 (real-time pcr) and #10 (molecular typing) appeared to serve as methodological bridges linking distinct research domains. These cross-cluster links point to several clinically and microbiologically relevant diagnostic issues. The etiology–RT-PCR linkage can be interpreted by considering a fundamental diagnostic ambiguity: whether detection of MP nucleic acid represents clinically relevant infection, asymptomatic carriage, or persistent detection following a recent infection; previous pediatric evidence has shown that conventional serology, quantitative PCR, and culture may not reliably distinguish carriage from symptomatic infection [48]. Among children with MPP, quantitative MP burden may further inform the assessment of disease severity and treatment response, with higher ddPCR-measured loads in severe MPP and marked declines after macrolide treatment [16]. The molecular-typing and macrolide-resistance themes further highlight the potential clinical value of molecular characterization, particularly the detection of macrolide-resistance determinants, beyond pathogen detection alone [36,43]. Relatedly, the connections among Cluster #10 (molecular typing), Cluster #3 (macrolide resistance), and Cluster #6 (refractory mycoplasma pneumoniae pneumonia) suggest a convergence of molecular typing, resistance profiling, and research on refractory clinical phenotypes [49]. Pathogen-oriented clusters, including Cluster #1 (streptococcal pneumonia) and Cluster #7 (chlamydia pneumoniae), are also consistent with the continuing need for differential diagnosis among respiratory pathogens with overlapping clinical presentations [50,51]. Collectively, these findings define a clinically oriented framework for MP diagnostics that integrates pathogen detection, molecular typing, and resistance profiling with differential diagnosis and the assessment of refractory phenotypes, disease severity, and treatment response.

### 4.4. Evolution of Research Hotspots and Transformation of Diagnostic Paradigms

Keyword analyses showed that PCR remains integral to MP diagnostic research. The continued use of serological terminology suggests that serology remains clinically relevant, particularly when molecular testing is not readily available or when understanding host immune responses can help determine infection timelines [52]. This coexistence suggests that MP diagnostics have not simply replaced traditional methods with advanced technologies, they have rather evolved into a multifaceted framework in which various tests play complementary roles. Within this structure, nucleic acid amplification techniques provide rapid pathogen-specific evidence, whereas serological assays contribute complementary information regarding host immune responses [40,53].

The emergence of molecular typing and resistance-related keywords further underscores the growing expectation that MP diagnostics will encompass strain characterization and resistance assessment [36,54]. More recently, “mNGS,” “tNGS,” and “co-infection” point to growing interest in broader multi-pathogen assessment, whereas “nomogram,” “risk factor,” and “RMPP” indicate increasing attention to clinical stratification.

Importantly, research priorities were not inferred from keyword frequency alone. In this study, themes were considered to be more strongly supported when the emergence and temporal evolution of recent keywords were consistent with the reference co-citation cluster structure. When viewed longitudinally, these patterns indicate a progressive shift from pathogen detection alone toward more clinically integrated diagnostic research. Accordingly, three key current research directions were identified: rapid and broader-spectrum pathogen detection; resistance-informed diagnosis; and risk identification, stratification, and management of refractory or severe MPP.

Future diagnostic research should, therefore, assess diagnostic tools in terms of sensitivity, specificity, timeliness, resistance profiling, cost-effectiveness, clinical interpretability, and utility in guiding treatment decisions.

These current hotspots also provide insight into potential contributions from emerging technologies. Novel modalities such as RPA-CRISPR/Cas12a, artificial intelligence, and nanomaterial-based assays could enhance rapid testing, automate result interpretation, and improve detection sensitivity for MP diagnosis. Their limited representation in clustering analyses should be interpreted cautiously, as it may reflect early stages of clinical translation, heterogeneous terminology, or insufficiently cited evidence rather than diminished scientific value. Future studies should investigate whether these approaches elicit improvements in clinically meaningful outcomes, including earlier diagnosis, optimal antimicrobial use, reduction in unnecessary testing, and more accurate identification of individuals at elevated risk for severe or refractory disease.

### 4.5. Disease-Term Analysis and Future Research Directions

Disease-term analysis provided a clinical perspective on the evolving landscape of MP diagnostic research. High-frequency terms such as MPP, CAP, and bacterial pneumonia reflect the traditional emphasis on respiratory infections and etiological differentiation. More recent attention to pleural effusion, pulmonary atelectasis, respiratory distress syndrome, co-infection, and refractory pneumonia suggests that current studies have increasingly addressed disease progression, complications, and poor treatment responses. This trend suggests that the focus of MP diagnosis has expanded beyond pathogen identification to encompass clinically relevant phenotypes, disease severity, and complications.

However, these disease-term associations should be interpreted with caution. Co-occurrence within a bibliometric network does not inherently establish clinical association, causality, or comorbidity. Although previous studies have described a potential relationship between MP infection and communication disorders [55,56], confirmation of this relationship requires targeted clinical and mechanistic investigations, rather than inference from co-occurrence data alone.

From a clinical perspective, these observations underscore the need for future diagnostic research to focus on patients presenting with complicated or atypical disease courses. Given that MP infection may involve pulmonary and extrapulmonary manifestations and that disease severity may be affected by factors such as immune-mediated injury, inflammation, pathogen burden, co-infections, vascular events, and delayed diagnosis or ineffective treatment [26,57], pathogen detection alone may be insufficient for comprehensive clinical assessment in cases of refractory or severe disease. Future research should prioritize the development and validation of integrated diagnostic algorithms that incorporate molecular testing, resistance markers, inflammatory biomarkers, imaging features, and validated clinical risk scores to support risk assessment and individualized patient management.

## 5. Limitations

This study has certain limitations. First, only English-language original articles from the WoSCC were included; hence, studies reposited in other databases, such as PubMed, Scopus, and Embase, may have been missed. The selective journal coverage of the WoSCC and the restriction to English-language publications may also underrepresent research from certain countries or regions. In addition, citation counts and citation-based network measures may vary across databases owing to differences in citation indexing and source coverage. Second, the search strategy was relatively concise to balance retrieval sensitivity and specificity, which may not capture all relevant terminology or technology-specific terms used in MP diagnostics, such as “assay,” “identification,” “screening,” “molecular detection,” “serology,” or “point-of-care testing.” Third, the literature search was completed on 12 May 2025, resulting in partial-year data for 2025, which could affect the interpretation of recent trends. Finally, bibliometric indicators reflect publication activity and network structure, but do not directly assess diagnostic accuracy, clinical effectiveness, or patient outcomes. Accordingly, future research should integrate multi-database bibliometric analysis, systematic reviews, diagnostic-accuracy meta-analyses, and clinical validation to ensure more comprehensive evaluation.

## 6. Conclusions

This study provided a bibliometric analysis of diagnostic research on MP from 1980 to 2025, mapping its structural evolution and emerging priorities. The findings indicate that MP diagnosis has evolved from traditional serology and pathogen confirmation toward a more integrated framework involving molecular methods, resistance assessment, host-response analysis, and clinical risk prediction. PCR and molecular typing remain central methodological pillars, supporting the transition from isolated pathogen detection to more clinically actionable diagnostic strategies. These insights clarify the current knowledge structure, research frontiers, and unmet needs in MP diagnostics. Future research should prioritize the development of rapid and accessible diagnostic platforms, integration of molecular and host-response markers, external validation of risk prediction models, and large-scale international collaboration to standardize diagnostic procedures and resistance surveillance frameworks. Such efforts could lead to more accurate, accessible, and clinically informative MP diagnostics.

## Figures and Tables

**Table 1:** Top 20 countries/regions by publication output.

Rank	Countries/Regions	Number of Publications	Total Link Strength	Citations	Average Citations
1	China	623	60	8348	13.40
2	United States	387	138	21,143	54.63
3	Japan	138	30	3021	21.89
4	Germany	109	117	4606	42.26
5	United Kingdom	80	100	4538	56.73
6	Finland	77	19	4677	60.74
7	Netherlands	75	87	3899	51.99
8	France	74	90	4618	62.41
9	South Korea	66	15	1390	21.06
10	Spain	60	84	3066	51.10
11	Italy	55	43	2355	42.82
12	Sweden	53	58	2792	52.68
13	Australia	50	47	3951	79.02
14	India	47	7	676	14.38
15	Belgium	42	82	2248	53.52
16	Denmark	37	63	1576	42.59
17	Israel	37	41	1206	32.59
18	Switzerland	37	40	1108	29.95
19	Canada	32	36	1853	57.91
20	Austria	29	37	575	19.83

## Data Availability

The original contributions presented in this study are included in the article/Supplementary Materials. Further inquiries can be directed to the corresponding authors.

## Supplementary Materials

The following supporting information can be downloaded at: https://www.mdpi.com/article/10.3390/pathogens15090912/s1, Figure S1. Country/region publication trends and citation bursts; Figure S2. Burst analysis of institutions, journals, references, and keywords; Figure S3. Top 10 authors with the strongest citation bursts and network of co-cited references; Table S1. Top 20 country/region collaborative pairs by normalized link strength; Table S2. Top 10 most productive institutions and top 10 institutional collaborative pairs by normalized link strength; Table S3. Top 20 journals by publication output; Table S4. Top 10 most productive authors and top 10 author collaborative pairs by normalized link strength; Table S5. Top 10 keywords by frequency.

## Abbreviations

The following abbreviations are used in this manuscript:

CAP	community-acquired pneumonia
CAGR	compound annual growth rate
CDC	Centers for Disease Control and Prevention
CRISPR	clustered regularly interspaced short palindromic repeats
CT	computed tomography
LAMP	loop-mediated isothermal amplification
mNGS	metagenomic next-generation sequencing
MP	*Mycoplasma pneumoniae*
MPP	*Mycoplasma pneumoniae* pneumonia
NIH	National Institutes of Health
PCR	polymerase chain reaction
RMPP	refractory *Mycoplasma pneumoniae* pneumonia
RPA	recombinase polymerase amplification
RT-PCR	real-time polymerase chain reaction
TLS	total link strength
tNGS	targeted next-generation sequencing
WoSCC	Web of Science Core Collection
